# Biology of *Meccus pallidipennis* (Hemiptera: Reduviidae) to Other Conditions Than That Encountered in Their Native Habitat

**Published:** 2018-09-30

**Authors:** Edson Franzim-Junior, Maria Tays Mendes, Ana Carolina Borella Marfil Anhê, Thiago Alvares da Costa, Marcos Vinicius Silva, César Gómez Hernandez, Afonso Pelli, Helioswilton Sales-Campos, Carlo Jose Freire Oliveira

**Affiliations:** 1 Instituto de Ciências Biológicas e Naturais, Universidade Federal do Triângulo Mineiro, Uberaba, Minas Gerais, Brazil; 2 Instituto de Tecnologia e Ciências Exatas, Universidade Federal do Triângulo Mineiro, Uberaba, Minas Gerais, Brazil

**Keywords:** Behavior, Biology, Hemiptera, *Meccus pallidipennis*, Triatominae

## Abstract

**Background:**

*Meccus pallidipennis* (Hemiptera: Reduviidae) is only found in Mexico and is one of the most important vectors for *Trypanosoma cruzi* transmission there. Because data concerning the ability of this bug to adapt to different environments are scarce, we aimed to elucidate its biology, behavior and ability to acclimatize to different environmental conditions.

**Methods:**

From the eclosion of 90 1^st^ instar nymphs, development was followed until the adult phase. Adults were fed after 30 days of fasting, and the average amount of blood ingested, the time between the beginning of the blood meal and the production of feces, and the frequency of stools/insect were recorded during their meals. After taking a blood meal, couples were isolated and monitored for 21 days, during which eggs were collected weekly.

**Results:**

The development of *M. pallidipennis* took 171.74±7.03 days to complete its life cycle, and females ingested larger amounts of blood than males. Oviposition was constant and did not demonstrate a significant decrease during this study.

**Conclusion:**

*Meccus pallidipennis* was able to acclimatize to fluctuating laboratorial conditions other than those naturally found in Mexico.

## Introduction

Chagas disease affects approximately 10 million people around the world, and at least 25 million are exposed to risk of infection (1). The protozoan *Trypanosoma cruzi* is the causative agent, and the most frequent form of transmission is through the blood-sucking arthropods known as triatomines or “kissing bug” insects. After taking a blood meal, infected insects release feces contaminated with infectious meta-cyclic trypomastigotes adjacent to the site of the bite (2). Because insect vectors are the main route of transmission in endemic countries, the most effective strategy to restrain the spread of disease is to better understand the vector behavior and biology.

During their life cycle, triatomines go through five different nymphal stages before entering the adult phase when they reach their sexual maturity and are able to reproduce. Their biology is species-specific and depends on environmental conditions, such as temperature and humidity range (3). Additionally, aspects concerning food intake may also have an impact on their epidemiology. In this context, there is a positive relationship between food intake and feces production, which together with the times for feeding and evacuation, are key elements that dictate the vector efficiency and disease spread (4).

So far, more than 140 species of triatomines have been identified. Among them, *Meccus pallidipennis* Stål (Hemiptera: Reduviidae), formerly known as *Triatoma pallidipennis*, is primarily found on the Pacific coast of Mexico and represents one of the main vectors of Chagas disease in that country (5). This insect is found either in sylvan or domestic reservoirs (6). In urban areas, *M. pallidipennis* is associated with the presence of dogs, squirrels, pigs and opossums near houses and unconstructed areas (7). The environment seems to affect some crucial aspects concerning the development, reproduction and the potential of *M. pallidipennis* to transmit Chagas disease in Mexico (8).

Because of the epidemiological importance of *M. pallidipennis* in Mexico and the relevance of this vector to different environments, we aimed to elucidate the biological and behavioral aspects of this species under laboratorial fluctuating conditions of humidity and temperature. For this purpose, we investigated the biology and timing of the different stages of development, feeding behavior and oviposition.

## Materials and Methods

### Meccus pallidipennis

*Meccus pallidipenis* specimens were originated from the region of Cienega state of Jalisco, and gently donated by Dr José Alejandro Martínez Ibarra from Universidad de Guadalajara, Mexico. Then, the second generation of triatomines were raised and kept in small plastic flasks (6.0cm height×7.0cm diameter) until they reached the 4^th^ nymphal instar at the insectary of the Federal University of Triângulo Mineiro (UFTM). Older nymphs and adults were kept in intermediate containers (7.5cm height × 11.0cm diameter). During the period of the study, triatomines were submitted to a natural light cycle and fluctuating environmental conditions of temperature (max: 31.03±0.81 °C and min: 18.87±1.387 °C) and humidity (56.85± 25.66%) in Uberaba, Minas Gerais, Brazil (19° 44′ 52″ S, 47° 55′ 55″ W–823m above sea level). Specimens were fed on immobilized and anesthetized Swiss mice weekly. Mice were from the animal facility at the Parasitology Division of the UFTM in Uberaba, Minas Gerais, Brazil and were anesthetized by using sodium tyopenthal (40.0mg/kg intraperitoneal-i.p.).

All procedures were submitted and approved by the Institutional Animal Care and Use Committee of the Federal University of Triângulo Mineiro (Brazil) under protocol 307.

### Life cycle of *Meccus pallidipennis*

We aimed to demonstrate the egg-to-adult developmental time under the aforementioned environmental conditions. After oviposition, eggs were collected and monitored until eclosion. Then, 90 nymphs were divided in colonies containing 30 insects each, and the time to reach the adult phase was recorded. Triatomines in the 1^st^ nymphal stage were fed 10 days after eclosion (2^nd^ and 4^th^ nymphal stages) and were followed until the next molt. Insects in the 5^th^ nymphal stage were fed twice, on the 10^th^ and 20^th^ days after molting. Only nymphs that fed were considered viable and able to develop from one developmental stage to another, and 5^th^ instar nymphs were considered viable only after the second feeding.

### Feeding behavior

Feeding behavior analyses verified the average time of feeding, amount of blood ingested, and time between blood meal and excretion. During the blood meal, the following aspects were recorded: feeding time, amount of blood ingested, time until first excretion and frequency of feces. The frequency of feces/meal was also recorded. Randomly, 20 adult insects (10 males and 10 females), fasting for 28 days, were selected from pre-existing colonies. Each triatomine was individually allocated to plastic receptacles and weighed before and after the blood meal using an analytic scale (UniBloc Shimadzu AUY220, Kyoto, Japan). The blood ingestion rate was individually calculated by dividing the amount of blood ingested by the feeding duration. The amount of feces/meal was calculated by dividing the frequency of total defecations per gender by the number of triatomines from each group/gender. Both the blood ingestion rate and the amount of feces per meal were calculated as previously describe (9), with some modifications.

### Oviposition during different fasting times

The oviposition was followed during different fasting times to evaluate the impact of fasting on the production of eggs. Thus, 8 adult couples were randomly chosen, isolated, and fed only once, and oviposition was monitored daily over 21 days. Eggs were collected on days 7, 14 and 21 after feeding and kept in different containers for analysis.

### Statistics

For all variables, the normal distribution and homogeneous variance were tested. Owing the size of our groups, we used non-parametric Mann-Whitney tests to compare time of feeding, blood ingestion and blood ingestion rate. To address the differences between the percentage of males and females producing feces a Fisher exact test was used. Since we had only eight couples, a non-parametric method (Friedman test) was used to compare oviposition among weeks. The results were expressed as means±standard error of the means. The differences were considered statistically significant when P< 0.05 (5%). All analyses were performed using GraphPad Prism 5.0 software (San Diego, CA).

## Results

### *Meccus pallidipennis* developed under fluctuating environmental conditions

To study the impact of fluctuating environmental conditions on the development of *M. pallidipennis*, 90 nymphs were randomly selected, and their development was followed from hatching to adulthood. The mean time for eclosion was 20.15±0.74 days (Table 1). Furthermore, the number of days necessary to complete each instar increased when compared to the previous stage of development as follows: 22.13±0.83, 23.35±0.85, 25.13±1.03, 31.00± 1.44 and 50.00±2.60 days, respectively, from 1^st^ nymphal instar to adult (Table 1).

**Table 1.** Time of transition between developmental stages

**Developmental stage**	**Median±SD (days)**
**Eggs - N1 (n=80)**	20.15±0.74
**N1-N2 (n=56)**	22.13±0.83
**N2-N3 (n=49)**	23.35 ±0.85
**N3-N4 (n=39)**	25.13 ±1.03
**N4-N5 (n=27)**	31.00 ±1.44
**N5–Adult (n=6)**	50.00 ±2.60
**Total**	171.74 ±7.03

n: number of individuals in each developmental stage

### *Meccus pallidipennis* females ingested larger amount of blood than males

Because we observed that *M. pallidipennis* was able to properly develop under the local environmental conditions, we next assessed their feeding behavior. The results showed that females ingested larger amounts of blood than males (Table 2). No differences between genders were observed for the feeding duration and blood ingestion rate (Table 2). Furthermore, 70% of adult males defecated in 26.69±7.98 minutes after starting the blood meal and had 0.80±0.63 feces/meal (Table 3), while females had 1.30±0.48 feces/meal during 25.43±7.83 minutes (Table 3). Though slightly different, no significant differences were observed between the genders on these parameters.

**Table 2.** Feeding behavior in adult triatomines

	**Time of feeding (min)**	**Blood ingestion (mg)**	**Blood ingestion rate (mg/min)**
***M. pallidipennis (M)***	26.24±11.10	445.60±265.00 *	18.66±9.71
***M. pallidipennis (F)***	34.09±14.05	760.40±317.00 *	23.28±6.05

M: adult males. F: adult females. Min: Minute. mg: milligram. mg/min: milligram per minute. Number of individuals/gender = 10. To compare time of feeding, blood ingestion and blood ingestion rate a non-parametric Mann-Whitney test was used. To address the differences between the percentage of males and females producing feces a Fisher exact test was used. Data are depicted as mean±standard deviations. An asterisk (*) indicates a significant result (P< 0.05)

**Table 3.** Production of feces

	**Time of defecation (min)**	**% insects producing feces**	**Frequency of defecation/meal**
***M. pallidipennis* (M)**	29.69±7.98	70	0.80±0.63
***M. pallidipennis* (F)**	25.43±7.83	100	1.30±0.48

M: adult males. F: adult females. Min: minute. Number of individuals/gender = 10. Data are depicted as mean±standard deviations

### Oviposition

**Fig. 1.**
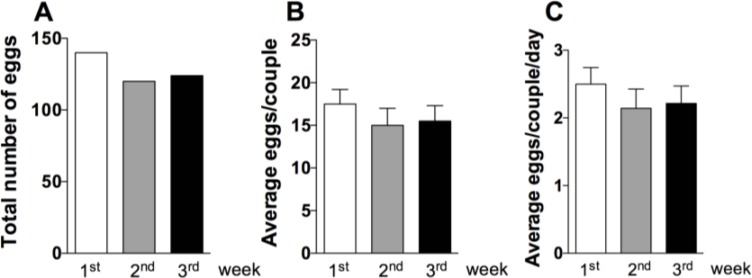
Oviposition of *Meccus pallidipennis* during fasting period After a blood meal, eight couples were separated into individual plastic flasks for 3 weeks as previously described. (A) Total number of eggs; (B) Eggs per couple/week and (C) Average eggs per couple per day. To compare the differences in oviposition among weeks a Friedman test was used. The results were expressed as means±standard error of the means.

Because triatomines were able to develop and fed under the conditions tested, we next investigated their ability to lay eggs. Overall, 140, 120 and 122 eggs were laid during the first, second and third week, respectively (Fig. 1a). Weekly, the oviposition rate was 17.50±4.81, 15.00±5.63 and 15.50±5.09, respectively, from the first to third week (Fig. 1b). Furthermore, daily oviposition was recorded, and the rates were 2.50±0.68, 2.14±0.80 and 2.21±0.72, respectively, for the first, second and third week (Fig.1c). However, differences among the weeks were not significant.

## Discussion

*Meccus pallidipennis* was able to acclimatize to environmental conditions other than those naturally found in Mexico, reinforcing its vector potential in a distant geographical area. In this study, the life cycle of *M. pallidipennis* was similar to that observed in previous studies with the same species (10, 11), despite the differences in food sources and environmental conditions observed in the different studies. Notably, this insect has the shortest biological cycle among triatomines of the same genre (12, 13); however, even under the same laboratory conditions, life cycles can be distinct in different populations from the same species (14). The heterogeneity of the populations of *M. pallidipennis* was further demonstrate in a study that showed the impact of different environments on the reproduction, feeding behavior and development of this species in distinct areas of Mexico (8). Short biological cycles are epidemiologically critical because they represent the ability of a species to repopulate and reestablish colonies right after disturbances, such as those produced by insecticides. This scenario makes triatomines more competent and capable of interacting with a host, consequently increasing the risk of transmission of *T. cruzi*. When compared to the main species of vectors in Latin America, the biological cycle of *M. pallidipennis* was longer than that described for *T. infestans* and *Rhodnius prolixus*, both reaching adult stage within less than 100 days (15, 16). However, some other species may have a longer life cycle, such as that observed in *T. boliviana* (252 days), from Bolivia (17), and *T. carcavalloi* (193 days), from the Southern region of Brazil (18). On the other hand, it was similar to the development of *Panstrongylus megistus* (19) and *Eratyrus mucronatus* (20).

Feeding behavior is one of the key elements that dictate the vector efficiency and biology of triatomines. During a blood meal, not only they are able to transmit *T. cruzi* but also to fulfill their physiological needs for survival. Females are more sensitive to fasting periods than males from the same species (21). Different factors could explain this characteristic, including the energy expended in oviposition and the maintenance of larger bodies in adult females compared to their male counterparts. Accordingly, we demonstrated that adult females ingested greater amounts of blood than males, which could be related to the higher physiological and energetic demand associated with females. However, no differences between genders were observed for the feeding duration and rate of blood ingestion. Though the feeding duration for females was similar to that observed in males, the higher amount of blood ingested by females may suggest a greater voracity to their hosts. The observation that females ingest higher amounts of blood than males could explain why they are also considered better vectors, besides as a mechanism for greater feces production (9, 14). Unfortunately, we did not find any differences between the amount of blood ingested and the production of feces when the genders were compared. Additionally, because a positive relationship was already established between the influence of the fasting period and the production of feces (22), we believe the fasting period in our study (30 days) might have increased the time needed for defecation. Furthermore, as females are more sensitive to fasting, they also tend to be more sensitive to weight loss when compared to their male counterparts from the same species. This characteristic makes females less-efficient vectors when exposed to greater fasting periods. Despite the impact on production of feces, the fasting period did not seem to influence the proportion of insects defecating before or after feeding. In the absence of regular feeding periods, either oviposition or fertility could be negatively influenced (23).

However, in our study, fasting did not influence oviposition. These results indicate that the period of and sensitivity to fasting may fluctuate according to the feeding behavior, and longer feeding periods seem to enhance the period of oviposition.

## Conclusion

Feeding behavior has a great impact on the life cycle of triatomines, which may also influence *T. cruzi* transmission. Furthermore, the higher amount of blood ingested by females than males reinforced their potential as vectors. Finally, because *M. pallidipennis* was able to reach sexual maturity and reproduce even under different and fluctuating environmental conditions, it seems reasonable to assume its potential as a vector in environments other than those naturally find in Mexico.
